# The circRAB3IP Mediated by eIF4A3 and LEF1 Contributes to Enzalutamide Resistance in Prostate Cancer by Targeting miR-133a-3p/miR-133b/SGK1 Pathway

**DOI:** 10.3389/fonc.2021.752573

**Published:** 2021-11-15

**Authors:** Dong Chen, Yaqin Wang, Feiya Yang, Adili Keranmu, Qingxin Zhao, Liyuan Wu, Sujun Han, Nianzeng Xing

**Affiliations:** ^1^ Department of Urology, National Cancer Center/National Clinical Research Center for Cancer/Cancer Hospital, Chinese Academy of Medical Sciences and Peking Union Medical College, Beijing, China; ^2^ State Key Laboratory of Molecular Oncology, National Cancer Center/National Clinical Research Center for Cancer/Cancer Hospital, Chinese Academy of Medical Sciences and Peking Union Medical College, Beijing, China; ^3^ Key Laboratory of Cardiovascular Epidemiology and Department of Epidemiology, Fuwai Hospital, National Center for Cardiovascular Diseases, Chinese Academy of Medical Sciences and Peking Union Medical College, Beijing, China

**Keywords:** circRAB3IP, eIF4A3, LEF1, SGK1, enzalutamide resistance

## Abstract

An increasing number of studies have shown that circRNAs are closely related to the carcinogenesis and development of prostate cancer (PCa). However, little is known about the effect of the biological functions of circRNAs on the enzalutamide resistance of PCa. Through bioinformatic analysis and experiments, we investigated the expression pattern of circRNAs in enzalutamide-resistant PCa cells. Quantitative real-time PCR was used to detect the expression of circRAB3IP, and plasmids that knock down or overexpress circRAB3IP were used to evaluate its effect on the enzalutamide sensitivity of PCa cells. Mechanistically, we explored the potential regulatory effects of eIF4A3 and LEF1 on the biogenesis of circRAB3IP. Our *in vivo* and *in vitro* data indicated that increased expression of circRAB3IP was found in enzalutamide-resistant PCa, and knockdown of circRAB3IP significantly enhanced enzalutamide sensitivity in PCa cells. However, upregulation of circRAB3IP resulted in the opposite effects. Further mechanistic research demonstrated that circRAB3IP could regulate the expression of serum and glucocorticoid-regulated kinase 1 (SGK1) by serving as a sponge that directly targets miR-133a-3p/miR-133b. Then, we showed that circRAB3IP partially exerted its biological functions *via* SGK1 signaling. Furthermore, we discovered that eIF4A3 and LEF1 might increase circRAB3IP expression in PCa.

## Introduction

In 2018, prostate cancer (PCa) was the second most frequently diagnosed cancer and the fifth leading cause of cancer deaths in men worldwide (1). In 2020, PCa was responsible for almost 191,930 new cases and an estimated 33,330 associated deaths, making it the most frequently diagnosed cancer and the second leading cause of cancer deaths in the United States (2).

Enzalutamide (Enz, also named MDV3100) is the first second-generation AR antagonist with an 8-fold higher affinity for AR than bicalutamide and has shown significant efficacies in the clinic when used as first-line therapy for patients with metastatic prostate cancer in combination with androgen deprivation therapy (ADT) (3–5). Thus, it has been approved by the US Food and Drug Administration (FDA) for the treatment of metastatic and nonmetastatic castration-sensitive prostate cancer (CRPC), as well as metastatic castration-sensitive prostate cancer (mCSPC). Studies on the mechanism of Enz showed that it could suppress multiple steps of the AR signaling pathway, including androgen binding to AR, nuclear translocation of activated AR and coactivator recruitment, which induces cell apoptosis while inhibiting the proliferation of PCa cells (6, 7). Unfortunately, despite the initial therapeutic effect of Enz, nearly all treated patients eventually develop resistance (8). Therefore, it is of high importance to understand this mechanism and explore new strategies to resolve resistance to Enz.

Circular RNAs (circRNAs) are highly conserved, stable, covalently closed RNA transcripts generated by back-splicing of a single pre-mRNA and have gene-regulatory potential (9, 10). Emerging evidence shows that circRNAs are closely related to many human diseases, especially cancers, and may serve as valuable biomarkers due to their abundance and stability (11, 12). Recent studies have reported widespread expression of circRNAs in PCa samples, and circRNAs may regulate the proliferation, migration, invasion and metastasis of PCa cells (13–19). Moreover, a previous study demonstrated that circRNAs are differentially expressed in enzalutamide-resistant (EnzR) PCa cells and are potentially associated with resistance to Enz (20). However, the detailed mechanisms underlying the role of circRNAs in EnzR PCa cells remain unclear.

Here, we found that a RAB3IP-derived circRNA, termed circRAB3IP, is highly expressed in EnzR PCa cells and that increased circRAB3IP may decrease Enz sensitivity by acting as a competing endogenous RNA (ceRNA) for miR-133a-3p/miR-133b to regulate serum and glucocorticoid-regulated kinase 1 (SGK1) expression. Further mechanistic research demonstrated that eIF4A3 and LEF1 might promote the biogenesis of circRAB3IP. More importantly, targeting SGK1 with its specific inhibitor GSK650394 restored Enz sensitivity to further suppress EnzR cell growth. Together, the results from these preclinical studies suggest that targeting this newly identified circRAB3IP/miR-133a-3p/miR-133b/SGK1 signaling pathway may help in the development of a better therapy to restore the Enz sensitivity of PCa cells.

## Materials and Methods

### Cell Lines, Culture and Transfection

The 22Rv1 cell lines were purchased from Shanghai Institutes for Biological Sciences. The PCa cell lines (C4-2, C4-2-EnzR, LNCaP and LNCaP-EnzR) were kindly provided by Prof. Chawnshang Chang (University of Rochester Medical Center). To establish ENZ resistant cell lines, C4-2 and LNCaP cells were cultured in medium containing ENZ. Concentrations of ENZ were gradually increased to 40 μM (from 10 μM to 40 μM for 6 months) for C4-2 cell lines and 20 μM ENZ (from 5 μM to 20 μM for 6 months) for LNCaP cell lines, while the parental cell lines were propagated in dimethyl sulfoxide as the vehicle for the same time period. All the PCa cell lines cultured in RPMI-1640 medium (Corning, NY, USA) supplemented with 10% FBS (Corning, NY, USA), penicillin/streptomycin (1:100; HyClone, UT, USA) and placed in an in humidified incubator containing 5% CO2 at 37°C. For cell transfection, cell lines at 70%–80% confluence were planted in 6-well plates.

The pLKO.1-circRAB3IP#1 and circRAB3IP#2, pLKO.1-eIF4A3, pLKO.1-LEF1, pLKO.1-shSGK1, pWPI-circRAB3IP, pWPI-eIF4A3, pWPI-LEF1, and pWPI-SGK1 plasmids, the psPAX2 packaging plasmid and pMD2G envelope plasmid (lentivirus: packaging: envelope, 2:1:1) were transfected into 293T cells using the standard calcium chloride transfection method for 48 h to obtain the lentiviral supernatant. miR-133a-3p/miR-133b mimics or inhibitors were designed and synthesized by RiboBio (Guangzhou, China). Lipofectamine 3000 (Invitrogen, CA, USA) was used for transfection following the standard method. 48 h later, cells were reaped for subsequent experiments.

### RNA Extraction and Real-Time PCR (qRT-PCR) Assay

The RNA extraction was performed according to the manufacturer’s instructions. Cells were collected and total RNA was extracted using TRIzol reagent (Invitrogen, CA, USA) and cDNA synthesis was performed using PrimeScriptRT reagent kit (Takara, Kusatsu, Japan). Real-time PCR was conducted using a Bio-Rad CFX96 system with SYBR green to determine the mRNA expression level of the gene of interest. 2^−ΔΔ^Ct method was used to calculate the relative expression of RNAs and GAPDH was used as the internal control. The primers used to amplify circRNAs and mRNA transcripts were synthesized by Sangon Biotech (Shanghai, China). The sequences of the primers are listed in **Table S1**.

### Cell Proliferation Assay

The transfected cells were seeded in 24-well plates (2 × 10^3^ cells per well) and cultured for 1, 3, 5, and 7 days. Cells were harvested and incubated with yellow tetrazolium MTT (3-(4, 5-dimethylthiazolyl-2)-2,5-diphenyltetrazolium bromide agent at 37 °C for 15 min and then dissolving in DMSO. The OD570 were calculated and recorded at a wavelength of 570 nm using a microplate reader instrument (Bio-Rad Laboratories, CA, USA).

### Western Blotting (WB) Assay

Briefly, cells were washed twice with cold PBS and lysed in RIPA lysis buffer. After measurement of protein concentrations, equal amounts of protein lysates (30 µg) were separated by the 10% SDS-PAGE gels, then transferred onto polyvinylidene difluoride membranes (Millipore, MA, USA). After PVDF membranes were incubated with 5% skim milk for 2 hours, they were sequentially incubated with primary antibodies at 4°C overnight, HRP-conjugated secondary antibodies for 1 h at room temperature, and visualized using an ECL system (Thermo Fisher Scientific). The primary antibodies used in the study were listed below: SGK1 (#ab43606; Abcam, Cambridge, UK), AGO2(#sc-376696; Santa Cruz, TX, USA), PREX1(#GTX128035; GeneTex, CA, USA), HOXA9(#sc-81291; Santa Cruz, TX, USA), PTPRK(#sc-374315; Santa Cruz, TX, USA), eIF4A3(#ab32485; Abcam, Cambridge, UK), LEF1 (#ab137872; Abcam, Cambridge, UK), PAX2(#ab32087; Abcam, Cambridge, UK) and GAPDH (#sc-166574; Santa Cruz, TX, USA).

### RNA Immunoprecipitation Assay

PCa cells were lysed in RNA immunoprecipitation (RIP) lysis buffer supplemented with RNase inhibitor and the cell extract were incubated with magnetic beads conjugated with anti-AGO2 antibody or non-specific anti-IgG antibody (EMD Millipore) with rotation at 4°C overnight. The RNA/antibody complex was washed three times with RIP buffer supplemented with RNase inhibitor and Proteinase K. The RNA was extracted using Trizol according to the manufacturer’s protocol and was subjected to qRT-PCR analysis.

### Biotin-Coupled miRNA Capture Assay

The specific biotin-labeled circRAB3IP and the control probes were synthesized by GenePharma (Shanghai, China). In brief, biotin-labeled circRAB3IP and control probes were transfected into PCa cells at a final concentration of 20 nmol/L overnight. Then, the cells were harvested The biotin-coupled RNA complex was then pulled down by incubating the cell lysate with streptavidin-coated magnetic beads (Thermo Fisher Scientific) at 4°C overnight. Subsequently, RNA was eluted and extracted using Trizol Reagent, and then evaluated by qRT-PCR analysis.

### RNA-FISH

RNA-FISH was carried out according to the manufacturer’s instructions using fluorescence-conjugated probes and FISH Kit supplied from RiboBio (Guangzhou, PR China). Briefly, cells were incubated with hybridization solution overnight at 37°C in the dark containing circRAB3IP probes. After strict wash in saline sodium citrate buffer, 4′,6-diamidino-2-phenylindole (DAPI, Beyotime Biotechnology, China) was used for nucleus staining. Fluorescent images were acquired with an Olympus FV10 laser scanning confocal microscope (Olympus, Tokyo, Japan).

### Chromatin Immunoprecipitation (ChIP) Assay

Cells were cross-linked with 4% formaldehyde for 10 min followed by cell collection and sonication with a predetermined power to yield genomic DNA fragments 300-1000bp in length. Lysates were precleared sequentially with normal rabbit IgG (#sc-2027, Santa Cruz Biotechnology) and protein A-agarose. Anti-LEF1 antibody (2.0 µg) was added to the cell lysates and incubated at 4°C overnight. As a negative control, IgG was used in the reaction. Specific primer sets were designed to amplify a target sequence within the human RAB3IP gene promoter. PCR products were analyzed by agarose gel electrophoresis.

### Actinomycin D Assay

PCa cells were seeded at 1 × 10^5^ cells per well in a 6-well plate overnight and then exposed to 2 mg/L actinomycin D (Sigma, USA) for 6, 12, 18 and 24 hours. The cells were harvested at the indicated time points and the stability of circRAB3IP was analyzed using qRT-PCR. Clinical samples and microarray.

### Human Tissue Samples

Patients underwent radical prostatectomy for PCa (n = 26) and benign prostatic hyperplasia (n = 10) underwent transurethral resection of the prostate at the Department of Urology, Beijing Chaoyang Hospital, China from July 2013 to October 2017. No treatment was administered prior to surgery. All the tissue specimens were confirmed by two experienced pathologists. Pathological grading was judged by Gleason points-scoring system, Gleason score > 8 (high PCa, n = 10) and Gleason score < 6 (low PCa, n = 10). The study protocol was approved by the Ethics Committee of Beijing Chaoyang Hospital and Verbal consent was obtained from each patient.

### Animal Experiments

All animal protocols were approved by the Animal Care and Use Committee of the Chinese Academy of Medical Sciences Cancer Hospital. Male BALB/c nude mice (4 weeks old) were maintained under specific pathogen-free conditions and manipulated according to protocols. C4-2-EnzR (1 × 10^6^) cells were mixed with Matrigel (1:1) and subcutaneously injected into the flanks of each mouse. once tumors reached 200 mm^3^, mice were randomly separated into four groups for different treatments. Tumor-bearing mice were randomized into four groups and treated by i.p. every other day as follows: (1) vehicle, (2) Enz (30 mg/kg), (3) GSK650394 (10 mg/kg) and (4) Enz (30 mg/kg) + GSK650394 (10 mg/kg). The tumor volumes were measured every 4 days with calipers and were calculated according to the following formula: tumor volume= (length × width^2^)/2.

### Statistical Analyses

All data were indicated as means ± standard error of the mean (SEM) processed by GraphPad Prism 8.0 (La Jolla, USA). Student’s t-test was used to analyze the differences between two groups and one-way ANOVA was used for multiple comparisons. A value of p <0.05 was considered statistically significant.

## Results

### Identification and Characterization of circRAB3IP in EnzR PCa Cells

**Figure 1:**
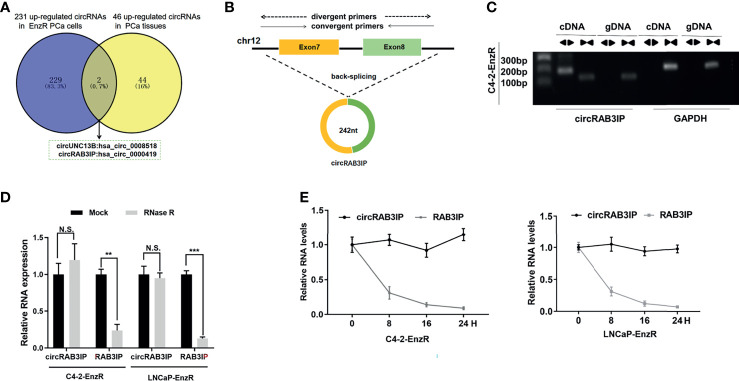
**(A)** Analysis of circRNA microarray data (GSE118959) from EnzR PCa cells and RNA-seq data of 25 pairs of matched tumor and normal for PRAD samples by Josh et al. **(B)** Schematic diagram of the genomic location and splicing pattern of circRAB3IP. The divergent and convergent primers were designed to detect circRAB3IP and RAB3IP, respectively. **(C)** PCR was performed to detect the existence of circRAB3IP from cDNA and gDNA in C4-2-EnzR cells using divergent and convergent primers, respectively. **(D)** qRT-PCR was conducted to determine the abundances of circRAB3IP and linear RAB3IP mRNA in PCa cells treated with RNase R at the indicated time points. **(E)** The RNA expression levels of circRAB3IP and RAB3IP were detected by qRT-PCR after treatment with actinomycin D at the indicated time points. Data are presented as mean ± SEM. **P < 0.01, ***P < 0.001 compared with the controls. N.S., not significant compared with the controls.

To identify the critical circRNAs involved in EnzR PCa, we analyzed published circRNA microarray data (GSE118959) from EnzR PCa cells (20) and RNA-seq data from 25 pairs of matched tumor and normal for localized primary prostate adenocarcinoma (PRAD) samples (17). The results showed that hsa_circ_0008518 and hsa_circ_0000419 were both highly enriched in PCa tissues and EnzR PCa cells (**Figure 1A**).

Assessment of the expression of these two circRNAs in EnzR PCa cell lines revealed that only hsa_circ_0000419 was significantly overexpressed compared to the parental PCa cell line (**Figures S1A, B**). Thus, we chose hsa_circ_0000419 as the target circRNA in this study. By browsing the human reference genome (GRCh37/hg19), we identified that hsa_circ_0000419 is located on chromosome 12 and derived from exons 7 and 8 of the RAB3IP gene; thus, we named it circRAB3IP (**Figure 1B**). To provide confirmation that circRAB3IP is differentially expressed in human PCa, prostatectomy specimens of patients with benign prostatic hyperplasia (BPH), low grade PCa (l-PCa, Gleason<6), high grade PCa (h-PCa, Gleason>8) and EnzR PCa samples were examined by qRT-PCR using divergent primers. Consistent with EnzR PCa cell lines results, circRAB3IP expression was significantly upregulated in EnzR PCa tissues compared with enzalutamide sensitive (EnzS) PCa tissues (**Figure S1C**). As expected, expression level of circRAB3IP was obviously higher in h-PCa tissues than in l-PCa or BPH tissues (**Figure S1C**). Moreover, we also found from another study that circRAB3IP was also significantly upregulated in h-PCa tissues compared with l-PCa tissues (**Figure S1D**) (19). Next, we also analyzed circRAB3IP expression by the MiOncoCirc database (https://mioncocirc.github.io), and the results indicated that circRAB3IP levels in prostate adenocarcinoma data were higher than those in other tumors (**Figure S1E**).

To verify the circular characteristics of circRAB3IP, we designed divergent primers to amplify the circRAB3IP form and convergent primers to amplify another exon of the linear RAB3IP mRNA. The PCR results showed that circRAB3IP was only detected in cDNA when using divergent primers in C4-2-EnzR cells, while no amplification product was obtained from gDNA (**Figure 1C**). Additionally, we found that circRAB3IP was resistant to digestion by RNase R compared with linear RAB3IP mRNA, further demonstrating that circRAB3IP exists as a circular RNA in PCa cells (**Figure 1D**). Finally, the results from an actinomycin D assay also indicated that the transcript half-life of circRAB3IP was more stable than that of its linear mRNA counterpart (**Figure 1E**).

### circRAB3IP Promotes Enzalutamide Resistance in PCa Cells

We designed two circRAB3IP shRNAs targeting the specific backsplice sequence (shcircRAB3IP#1 and shcircRAB3IP#2). The expression of circRAB3IP, but not linear RAB3IP mRNA expression, was successfully downregulated in C4-2-EnzR and LNCaP-EnzR cells (**Figures S2A, B**). Also, ectopic overexpression of circRAB3IP did not alter linear RAB3IP mRNA expression (**Figures S2C, D**).

**Figure 2:**
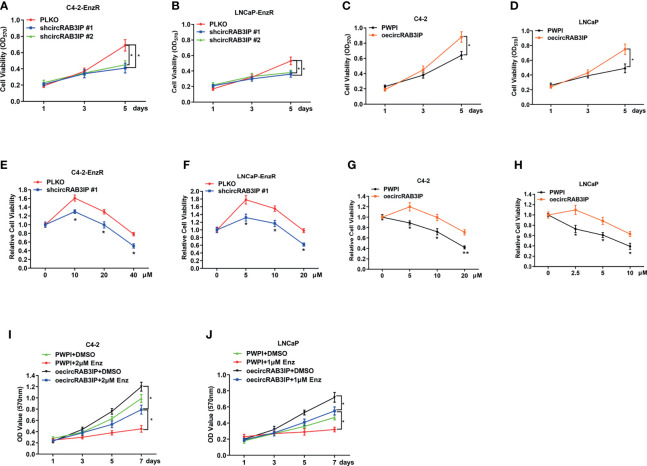
**(A)** The proliferation ability of C4-2-EnzR cells was evaluated by MTT assay after knocking down circRAB3IP. **(B)** The proliferation ability of LNCaP-EnzR cells was evaluated by MTT assay after knocking down circRAB3IP. **(C)** The proliferation ability of C4-2 cells was evaluated by MTT assay after overexpressing circRAB3IP. **(D)** The proliferation ability of LNCaP cells was evaluated by MTT assay after overexpressing circRAB3IP. **(E)** C4-2-EnzR cells were treated with different concentrations of Enz as indicated after knocking down circRAB3IP #1, and cell viability was evaluated by CCK-8 assay. **(F)** LNCaP-EnzR cells were treated with different concentrations of Enz as indicated after knocking down circRAB3IP #1, and cell viability was evaluated by CCK-8 assay. **(G)** C4-2 cells were treated with different concentrations of Enz as indicated after overexpressing circRAB3IP, and cell viability was evaluated by CCK-8 assay. **(H)** LNCaP cells were treated with different concentrations of Enz as indicated after overexpressing circRAB3IP, and cell viability was evaluated by CCK-8 assay. **(I)** C4-2 cells were treated with/without 1μM Enz after overexpressing circRAB3IP or not (PWPI), and cell viability was evaluated by CCK-8 assay. **(J)** LNCaP cells were treated with/without 2μM Enz after overexpressing circRAB3IP or not (PWPI), and cell viability was evaluated by CCK-8 assay. Data are presented as mean ± SEM. *P < 0.05, **P < 0.01 compared with the controls.

To link the increased circRAB3IP expression to Enz sensitivity in PCa cells, MTT assays showed that circRAB3IP knockdown could not induce a proliferation-suppressing effect on day 3 but could repress cell proliferation on day 5 (**Figures 2A–D**). Therefore, we chose 48 hours for MTT assays to test Enz sensitivity. Then, we treated C4-2-EnzR and LNCaP-EnzR cells with increased Enz doses for 48 hours to analyze Enz sensitivity when circRAB3IP was knocked down. These MTT assays indicated that knockdown of circRAB3IP promoted Enz sensitivity in EnzR PCa cells (**Figures 2E, F**, **S2E, F**) and that increased circRAB3IP expression led to the development of EnzR in C4-2 and LNCaP cells (**Figures 2G, H**). Moreover, overexpression of circRAB3IP in C4-2 and LNCaP cells significantly induced a proliferation-promoting effect, and this effect was partially inhibited by Enz (**Figures 2I, J**). Together, these data demonstrate that circRAB3IP could promote EnzR of PCa cells *in vitro*.

### circRAB3IP Directly Binds to miR-133a-3p and miR-133b in PCa Cells

**Figure 3:**
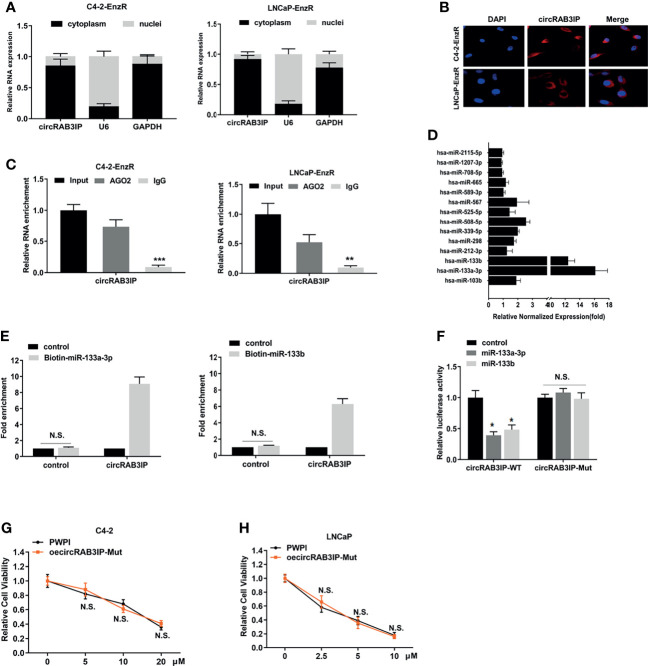
**(A)** Nuclear mass separation assays indicated the location of circRAB3IP, RAB3IP, U6 and GAPDH in C4-2-EnzR and LNCaP-EnzR cells. **(B)** RNA-FISH indicated the location of circRAB3IP in C4-2-EnzR and LNCaP-EnzR cells. **(C)** AGO2-RIP assays were performed using an antibody against AGO2 on extracts and detected by qRT-PCR assays in C4-2-EnzR and LNCaP-EnzR cells. **(D)** circRIP assays were performed in C4-2-EnzR cells using a circRAB3IP-specific probe, and the enrichment of miRNAs was detected by PCR. **(E)** A pull-down assay for biotin-labeled miRNA was used to evaluate the binding properties between miR-133a-3p/miR-133b and circRAB3IP in C4-2-EnzR cells. **(F)** A dual luciferase reporter assay was used to prove the binding properties between miR-133a-3p/miR-133b and circRAB3IP. **(G)** C4-2 cells were treated with different concentrations of Enz as indicated after overexpressing circRAB3IP-Mut or not (PWPI), and cell viability was evaluated by CCK-8 assay. **(H)** LNCaP cells were treated with different concentrations of Enz as indicated after overexpressing circRAB3IP-Mut or not (PWPI), and cell viability was evaluated by CCK-8 assay. Data are presented as mean ± SEM. *P < 0.05, **P < 0.01, ***P < 0.001 compared with the controls. N.S., not significant compared with the controls.

To further probe the underlying molecular mechanism of circRAB3IP in regulating Enz sensitivity, the subcellular localization of circRAB3IP was detected in EnzR PCa cell lines by nuclear-cytoplasmic fractionation assay and FISH assays. CircRAB3IP was predominantly distributed in the cytoplasm of EnzR PCa cells (**Figures 3A, B**).

The above results suggest that circRAB3IP may function at the posttranscriptional level and serve as a miRNA sponge in PCa cells. To confirm these predictions, we performed an AGO2-RIP assay in EnzR PCa cells, and the results revealed that circRAB3IP was significantly enriched by the AGO2 antibody (**Figure 3C**). These findings suggest that circRAB3IP might act as a binding platform for AGO2 and miRNAs.

Based on these findings, we predicted that circRAB3IP might serve as a binding platform for miRNAs in PCa cells. Then, we obtained miRNAs potentially binding circRAB3IP using the RegRNA database (21) (filter criteria: score ≥ 140 and free energy ≤ -20) and found 14 miRNA binding sites. Next, an RNA pull-down assay with a biotin-labeled circRAB3IP probe showed that circRAB3IP exhibited an adsorption affinity for miR-133a-3p and miR-133b in C4-2-EnzR cells (**Figure 3D**), and potential binding site analysis using the starBase v3.0 database is shown in **Figure S3A** (22).

As further confirmation of the binding of circRAB3IP with miR-133a-3p and miR-133b, the enrichment of circRAB3IP was significantly increased in both the biotin-labeled miR-133a-3p and miR-133b groups (**Figure 3E**). Subsequently, dual-luciferase reporter assays were executed to measure the binding between circRAB3IP and miR-133a-3p/miR-133b. The data indicated that both miR-133a-3p mimics and miR-133b mimics obviously reduced the luciferase activity of the circRAB3IP-WT luciferase reporter but not that of mutants (**Figure 3F**), which suggests that circRAB3IP might directly bind with both miR-133a-3p and miR-133b.

Moreover, circRAB3IP expression did not show significant changes after miR-133a-3p mimics or miR-133b mimics (**Figure S3B**), and expression of neither miR-133a-3p nor miR-133b showed significant changes after silencing circRAB3IP (**Figure S3C**). These findings suggest that circRAB3IP and miR-133a-3p/miR-133b may not be degraded by each other.

To prove that circRAB3IP promotes EnzR in PCa cells by sponging miR-133a-3p/miR-133b, we constructed a circRAB3IP vector with a mutated binding site of miR-133a-3p/miR-133b. The results from MTT assays revealed that mutation of the miR-133a-3p/miR-133b binding site could completely abolish circRAB3IP-decreased Enz sensitivity in C4-2 and LNCaP cells (**Figures 3G, H**).

Finally, we determined the expression levels of miR-133a-3p and miR-133b in TCGA-PRAD by starBase v3.0 database (22), and the results demonstrated that both miR-133a-3p and miR-133b were significantly downregulated in PCa (**Figure S3D**). Further analysis indicated that there were also positive associations between miR-133a/miR-133b expression and the pathology T stage (**Figure S3E**) and pathology N stage (**Figure S3F**).

### SGK1 Is a Direct Target of miR-133a-3p/miR-133b in PCa Cells

Given that circRAB3IP might act as a sponge for miR-133a-3p and miR-133b, we further explored the downstream targets of miR-133a-3p and miR-133b in PCa cells. We first performed bioinformatics analysis using the TargetScan, miRanda and PITA databases to identify the target genes of miR-133a-3p/miR-133b, and the results showed that there were 265 candidate genes that were predicted by all three databases. Next, to identify the upregulated genes in EnzR PCa cells, we analyzed published RNA-seq data (GSE104935) from EnzR PCa cells (23) (set the filter criteria as a fold-change ≥2). We found 786 upregulated genes in EnzR PCa cells, of which 13 were also the potential target genes of miR-133a-3p/miR-133b (**Figure S4A**). Moreover, we explored the OncoKB Cancer Gene List and found that 4 of them were cancer genes (24).

**Figure 4:**
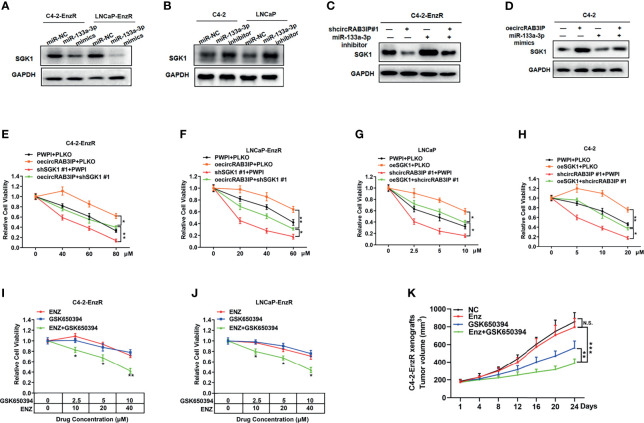
**(A)** The protein levels of SGK1 were detected by WB after treatment with miR-133a-3p mimics in C4-2-EnzR and LNCaP-EnzR cells. **(B)** The protein levels of SGK1 were detected by WB after treatment with miR-133a-3p inhibitors in C4-2 and LNCaP cells. **(C)** The upregulation of SGK1 in C4-2-EnzR cells transfected with miR-133a-3p inhibitors was reversed by knocking down circRAB3IP, as detected by WB. **(D)** The downregulation of SGK1 in C4-2 cells transfected with miR-133a-3p mimics was reversed by overexpression of circRAB3IP, as detected by WB. **(E)** C4-2-EnzR cells were treated with different concentrations of Enz as indicated after overexpressing circRAB3IP, knocking down SGK1 or their combination, and cell viability was evaluated by CCK-8 assay. **(F)** LNCaP-EnzR cells were treated with different concentrations of Enz as indicated after overexpressing circRAB3IP, knocking down SGK1 or their combination, and cell viability was evaluated by CCK-8 assay. **(G)** LNCaP cells were treated with different concentrations of Enz as indicated after overexpressing SGK1, knocking down circRAB3IP or their combination, and cell viability was evaluated by CCK-8 assay. **(H)** C4-2 cells were treated with different concentrations of Enz as indicated after overexpressing SGK1, knocking down circRAB3IP or both in combination, and cell viability was evaluated by CCK-8 assay. **(I)** C4-2-EnzR cells were treated with different concentrations of Enz, GSK650394 or their combination as indicated, and cell viability was evaluated by CCK-8 assay. **(J)** LNCaP-EnzR cells were treated with different concentrations of Enz, GSK650394 or their combination as indicated, and cell viability was evaluated by CCK-8 assay. **(K)** Mice bearing C4-2-EnzR xenografts were treated with control, Enz, GSK650394 or their combination for 24 days, and tumor volumes were measured every 4 days. Data are presented as mean ± SEM. *P < 0.05, **P < 0.01, ***P < 0.001 compared with the controls. N.S., not significant compared with the controls.

Next, WB analysis showed that among these 4 candidate cancer genes, only SGK1 was highly expressed in C4-2-EnzR cells (**Figures S4B, C**). In further experiments, we examined SGK1 protein expression in EnzR PCa samples (N=3) and EnzS PCa samples (N=3) by using WB and found that SGK1 protein levels were significantly lower in EnzS PCa samples than in EnzR PCa samples (**Figure S4D**). Thus, we investigated SGK1 as a potential target gene of miR-133a-3p/miR-133b, and the potential binding sites are shown in **Figure S4E**. To confirm our hypothesis, WB assays were performed and the results showed that the protein levels of SGK1 were markedly reduced in C4-2-EnzR and LNCaP-EnzR cells transfected with miR-133a-3p mimics (**Figure 4A**), while SGK1 expression were notably enhanced in C4-2 and LNCaP cells transfected with miR-133a-3p inhibitors (**Figure 4B**).

Further investigations suggested that knockdown of circRAB3IP decreased the protein levels of SGK1 in C4-2-EnzR cells, whereas overexpression of circRAB3IP resulted in the opposite effect in C4-2 cells. Importantly, these effects could be abolished by miR-133a-3p mimics or inhibitors (**Figures 4C, D**).

To further investigate the functional interaction between circRAB3IP and SGK1 in PCa cells, we designed two SGK1 shRNAs and one SGK1 ectopic expression plasmid. The silencing and overexpression efficiency of these plasmids were shown in **Figures S4F, G**. Next, rescue experiments were carried out by cotransfection of circRAB3IP-oe vector or shcircRAB3IP and SGK1-oe vector or shSGK1. The results revealed that overexpression of circRAB3IP significantly attenuated the Enz sensitivity promoting effects induced by downregulation of SGK1 in C4-2-EnzR and LNCaP-EnzR cells (**Figures 4E, F**). However, circRAB3IP knockdown could counteract the promoting roles of SGK1 overexpression in Enz sensitivity of C4-2 and LNCaP cells, as determined by MTT assays (**Figures 4G, H**). Moreover, we treated C4-2-EnzR and LNCaP-EnzR cells with the SGK1 inhibitor GSK650394, and the results from the MTT proliferation assay revealed that the viability of C4-2-EnzR and LNCaP-EnzR cells was not significantly reduced by GSK650394. Cotreatment with GSK650394 and Enz had a stronger inhibitory effect on cell viability than treatment with Enz alone (**Figures 4I, J**), suggesting restoration of Enz sensitivity in EnzR cells.

To test whether GSK650394 overcomes the Enz resistance of PCa *in vivo*, C4-2-EnzR xenografts were treated with vehicle, Enz, GSK650394 or their combination. As shown in **Figure 4K**, C4-2-EnzR xenografts were resistant to Enz treatment with tumor volumes comparable to those in the vehicle-treated control group. GSK650394 treatment alone decreased the tumor volume, while the combination of GSK650394 and Enz synergistically decreased C4-2-EnzR xenografts. These results indicate that GSK650394 could at least partially overcome Enz resistance and restore the Enz sensitivity of C4-2-EnzR xenografts *in vivo*.

### eIF4A3 and LEF1 Promote circRAB3IP Expression

**Figure 5:**
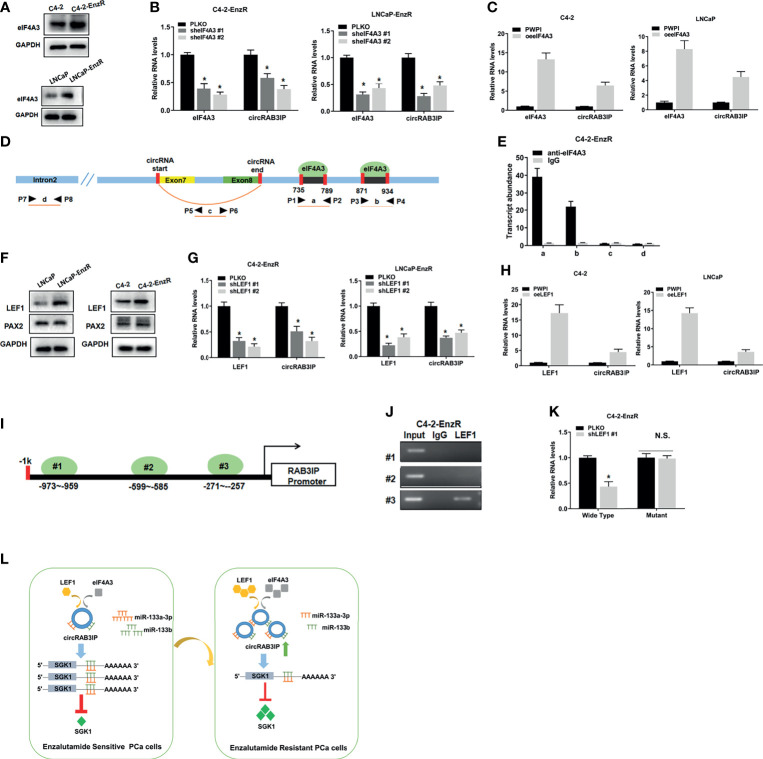
**(A)** The protein levels of eIF4A3 were detected by WB in PCa cells. **(B)** The expression of circRAB3IP was detected by RT-PCR after knocking down eIF4A3 in C4-2-EnzR and LNCaP-EnzR cells. **(C)** The expression of circRAB3IP was detected by RT-PCR after overexpressing eIF4A3 in C4-2 and LNCaP cells. **(D)** The putative binding sites of eIF4A3 in the upstream and downstream regions of the circRAB3IP pre-mRNA were predicted with the CircInteractome database. **(E)** RIP assay confirmed that eIF4A3 could directly bind to the RAB3IP pre-mRNA in C4-2-EnzR cells. **(F)** The protein levels of LEF1 and PAX2 were detected by WB in PCa cells. **(G)** The expression of circRAB3IP was detected by RT-PCR after knocking down LEF1 in C4-2-EnzR and LNCaP-EnzR cells. **(H)** The expression of circRAB3IP was detected by RT-PCR after overexpressing LEF1 in C4-2 and LNCaP cells. **(I)** Schematic illustration of three putative binding sites of LEF1 on the RAB3IP promoter is shown. **(J)** ChIP assays were performed to determine which putative LEF1 binding site the RAB3IP promoter was bound to in C4-2-EnzR cells. **(K)** Luciferase reporter assays were performed to determine whether LEF1 could promote the luciferase activity of the wild-type RAB3IP promoter. **(L)** The schematics of the mechanism which circRAB3IP might sponge miR-133a-3p and miR-133b to regulate SGK1 expression, leading to Enz resistance in PCa cells. Data are presented as mean ± SEM. *P < 0.05 compared with the controls. N.S., not significant compared with the controls.

We used the circInteractome database to explore the potential mechanism of circRAB3IP expression in PCa cells. We found that two possible binding sites of eIF4A3 are in the downstream region of the circRAB3IP mRNA transcript and might regulate circRAB3IP expression. WB results showed that eIF4A3 was highly expressed in EnzR PCa cells and samples (**Figures 5A** and **S5A**), and eIF4A3 overexpression or knockdown plasmids were synthesized (**Figures S5B, C**). To explore whether the expression of circRAB3IP could be regulated by eIF4A3, qRT-PCR analysis in C4-2-EnzR cells was carried out after transfection with eIF4A3 overexpression or knockdown plasmids. The results revealed that reducing the expression of eIF4A3 decreased circRAB3IP levels (**Figure 5B**), whereas upregulation of the expression of eIF4A3 significantly increased circRAB3IP levels (**Figure 5C**). Moreover, we defined the two potential eIF4A3 binding sites as A and B, the sequence of circRAB3IP as C, and the sequence of RAB3IP pre-mRNA intron 2 as D (**Figure 5D**). An RIP assay using anti-eIF4A3 antibody revealed that eIF4A3 could only bind to RAB3IP pre-mRNA through these two potential binding sites but not the sequence of circRAB3IP and RAB3IP pre-mRNA intron 2 (**Figure 5E**). The above results suggest that eIF4A3 might promote the expression of circRAB3IP in C4-2-EnzR cells.

Recent studies have shown that transcription factors (TFs) may promote the expression of circRNAs by binding to promoter regions. To determine whether upregulated TFs may promote circRAB3IP levels in C4-2-EnzR cells, we analyzed the PROMO database (25) and RNA-seq data from GSE104935. The results showed that 2 potential TFs within 1 kb of the promoter sequence were found (**Figure S5D**). Among these 2 potential TFs, WB results showed that only LEF1 was highly expressed in EnzR PCa cells and samples (**Figures 5F**, **S5E**), and LEF1 overexpression or knockdown plasmids were synthesized (**Figures S5F, G**). Then, qRT-PCR assays demonstrated that knockdown of LEF1 decreased circRAB3IP levels (**Figure 5G**), whereas upregulation of LEF1 significantly increased circRAB3IP levels (**Figure 5H**). To explore whether the expression of RAB3IP mRNA can be regulated by LEF1, we found 3 potential LEF1-binding sites on the RAB3IP promoter through the Jaspar database (26) (**Figure 5I**), and then ChIP assays performed with C4-2-EnzR cells revealed that LEF1 could only bind to the third binding site (**Figure 5J**). We then performed a luciferase reporter assay by inserting the 1 kb 5’ promoter region of RAB3IP containing the third binding site into the pGL3 luciferase backbone and generated a version with a mutated binding site (**Figure S5H**). The results revealed that knockdown of LEF1 significantly decreased luciferase activity in C4-2-EnzR cells transfected with the wild-type RAB3IP promoter construct but not in cells with the mutant RAB3IP promoter construct (**Figure 5K**). The above results demonstrate that LEF1 might transcriptionally regulate RAB3IP mRNA and increase circRAB3IP levels. Moreover, the expression levels of miR-133a-3p and miR-133b did not show significant changes knocking down or overexpressing eIF4A3 and LEF1 in EnzR PCa cells. (**Figures S5I, J**).Finally, we determined the expression levels of eIF4A3, LEF1 and RAB3IP in TCGA-PRAD from the GEPIA database (http://gepia.cancer-pku.cn/), UALCAN database and Linkedomics database (27, 28). The results demonstrated that both eIF4A3 and LEF1 were significantly upregulated in PCa (UALCAN database, **Figure S5K**), and there were also positive associations between both eIF4A3 and LEF1 expression and Gleason score (UALCAN database, **Figure S5L**), pathology T stage (Linkedomics database, **Figure S5M**) and pathology N stage (Linkedomics database, **Figure S5N**). Moreover, high expression of both eIF4A3 and LEF1 was associated with shorter disease-free survival (GEPIA database, **Figure S5O**).

## Discussion

In 2012, Enz was the first second-generation AR antagonist approved by the FDA for mCRPC patients after docetaxel treatment, and it significantly improved progression-free survival (29). Currently, it is the first FDA-approved antiandrogen to treat three forms of advanced PCa, including mCRPC, CRPC and mCSPC. However, the survival benefits of Enz were only achieved in approximately 50% of PCa patients, and almost all patients who initially responded to Enz treatment eventually developed resistance (6). There is an urgent need to determine the mechanisms of intrinsic and acquired resistance and could result in approaches to overcome such resistance. Previous studies have revealed some major mechanisms of acquired Enz resistance, including AR mutations and splice variants of AR, intratumoral androgen biosynthesis in PCa cells, lineage plasticity of PCa, cytokine dysregulation and the specific microenvironment of PCa (30–32). However, the accurate mechanisms of the resistance to Enz are still not clear.

Noncoding RNAs (ncRNAs), including miRNAs, lncRNAs, and circRNAs, are emerging as important regulators because of their crucial involvement in various biological processes. Recent studies have focused on circRNAs in cancer development. CircRNAs are a class of RNA molecules that lack 5’-3’ ends and poly A tails with high conservation and abundance, and they form closed continuous loops from exons or introns by back splicing or lariat formation (11). Compared with linear RNAs, circRNAs are more stable due to their higher resistance to exonuclease RNase R in cells, and they have higher expression in some tissues and body fluids (9). Previous studies have reported that circRNAs are dysregulated in PCa and play important roles in PCa initiation, development, and progression (13–19).

Recent studies have shown that circRNAs may also contribute to EnzR in PCa (20, 33). John et al. constructed EnzR PCa cells and screened circRNA expression in those cells using a circRNA microarray (20). They found that circRNAs are differentially expressed in EnzR PCa cells and are further altered depending on the extent of Enz resistance. Thus, their findings indicate that circRNAs are potentially associated with resistance to Enz and may potentially represent valuable prognostic biomarkers in the real-time monitoring of treatment response to Enz. However, the detailed mechanisms underlying the role of circRNAs in EnzR PCa cells remain unclear. In our study, we first analyzed circRNA microarray data and clinical PCa tissue data to assess the potential highly expressed circRNAs in both C4-2-EnzR cells (which may represent CRPC PCa cells) and clinical PCa samples. The results showed that circRAB3IP levels were significantly elevated in both C4-2-EnzR cells and PCa samples, and knocking down circRAB3IP expression increased the Enz sensitivity of EnzR cells. These results suggest that circRAB3IP expression may be associated with the development of EnzR in PCa cells. In this study, we report for the first time, to the best of our knowledge, circRAB3IP expression profiles associated with EnzR PCa; furthermore, no previous studies on circRAB3IP have been published.

To detect the potential mechanism of circRAB3IP in EnzR PCa cells, we first detected the cellular localization of circRAB3IP and determined that it was predominantly located in the cytoplasm. This indicates that circRAB3IP may function as a miRNA sponge, similar to competitive endogenous RNA molecules, and regulate gene expression at the posttranscriptional level. On this basis, we carried out bioinformatics analysis and mechanistic experiments to determine the downstream miRNAs of circRAB3IP. The results showed that circRAB3IP may exert its function as a ceRNA that competitively binds to miR-133a-3p and miR-133b and then abolishes the endogenous suppressive effect of these miRNAs on the target gene SGK1.

As previously reported, SGK1 is a member of the AGC kinase family of serine/threonine kinases and shares a large homologous sequence and kinase function with the AKT family (34). SGK1 expression is elevated in several tumors, including PCa, and is also associated with tumor growth, survival, cell cycle disorder, cancer stem cells, metastasis and chemoresistance (35, 36). Particularly, following androgen stimulation, both AR and glucocorticoid receptor (GR) activation could activate the response element motif region in the SGK1 promoter and upregulate SGK1 expression in PCa (37). Thus, we speculated that SGK1 expression may be linked to Enz sensitivity in PCa cells. From our results, we found that SGK1 protein levels were upregulated in EnzR PCa cells. We also found that loss-of-function of SGK1 by shRNAs or its specific inhibitor GSK650394 showed that SGK1 inhibition significantly increased Enz sensitivity in PCa cells and restored Enz sensitivity to further suppress EnzR PCa cell growth. Since GSK650394 has a sufficient safety and toxicity profile to advance to clinical trials, repurposing of this compound may represent an opportunity for rapid translation for clinical therapy of EnzR patients (38). Similarly, increased expression of SGK1 in EnzR LNCaP cell line models was observed by Massar et al., and inhibition of SGK1 using small molecular inhibitors significantly decreased the proliferation and migration of the EnzR LNCaP cell line (Abstract 316 from 2017 American Association for Cancer Research, DOI:10.1158/1538-7445.AM2017-316).

Several studies using cell lines have reported that miR-133a-3p and miR-133b are downregulated in PCa tissues and act as tumor-suppressive miRNAs that have been implicated in the development, progression and recurrence of PCa (39–41). Moreover, downregulation of miR-133b positively correlates with advanced pathological characteristics and shorter metastasis-free survival in PCa patients (41). circRAB3IP overexpression increased SGK1 expression, and knockdown of circRAB3IP reduced SGK1 expression. Thus, we speculated that the SGK1 gene might be a direct target of miR-133a-3p and miR-133b in PCa cells and that miR-133a-3p and miR-133b may restore Enz sensitivity in EnzR PCa cells. Similarly, previous studies also unveiled that SGK1 was the direct target of miR-133a-3p and miR-133b in other diseases (42–44).

Previous research has indicated that the biogenesis of circRNAs is mainly regulated by specific cis-acting elements or trans-acting factors, and it has been shown that certain transcription factors could also promote circRNA expression (9, 11). In this study, through bioinformatic analysis and experiments, we predicted and found that eIF4A3 and LEF1 can induce the formation of circRAB3IP. eIF4A3 is an ATP-dependent RNA helicase and the core component of the exon junction complex, which is an important component of mRNA splicing (45). In our study, we found that eIF4A3 could bind to the flanking sequence of circRAB3IP and regulate its expression. Moreover, previous studies have also shown that eIF4A3 could enhance circRNA expression combined with the flanking sequence of circRNAs, promoting its backsplicing (46–48). LEF1 is a transcription factor involved in many cancers, including PCa, and it belongs to the T-cell factor/LEF1 family that regulates gene expression by inducing structural alterations in the DNA helix (49). In PCa, LEF1 could promote AR expression and activity in an androgen-independent manner, ultimately increasing PCa growth and invasion regardless of androgen ablation therapy (50). In our study, we found that LEF1 could bind to the RAB3IP gene promoter region. We also showed that overexpression of LEF1 could increase the level of circRAB3IP and knockdown of LEF1 inhibited the expression of circRAB3IP. Moreover, Wang et al. demonstrated that LEF1 could upregulate circRNF121 expression by binding to the RNF121 promoter and enhancing RNF121 promoter activity (51).

In conclusion, we identified a novel circRNA, termed circRAB3IP, which is highly expressed in EnzR PCa cells and promotes PCa cell tolerance to Enz. Mechanistically, we found that circRAB3IP might sponge miR-133a-3p and miR-133b to regulate SGK1 expression, leading to Enz resistance in PCa cells. Targeting SGK1 with its inhibitor could restore the Enz sensitivity of PCa cells and is a potential target for Enz resistance. Moreover, we also demonstrate that eIF4A3 and LEF1 could mediate the biogenesis of circRAB3IP, but the detailed mechanisms in this regulatory process need further elucidation. Therefore, our study provides a solid basis for the development of a better understanding of Enz resistance pathology and for the identification of potential therapeutic drug targets for the treatment of Enz resistance (**Figure 5L**).

## Data Availability Statement

The original contributions presented in the study are included in the article/**Supplementary Material**. Further inquiries can be directed to the corresponding author.

## Ethics Statement

The studies involving human participants were reviewed and approved by the Cancer Hospital of Chinese Academy of Medical Sciences and Peking Union Medical College. The patients/participants provided their written informed consent to participate in this study. The animal study was reviewed and approved by the Cancer Hospital of Chinese Academy of Medical Sciences and Peking Union Medical College.

## Author Contributions

NX and SH conceived and designed the study. DC, YW, AK, QZ, and LW performed the experiments. DC, YW, FY, and AK conducted the statistical analyses. DC and YW wrote the paper, SH and NX revised the paper. All authors contributed to the article and approved the submitted version.

## Funding

This work was supported by the National Natural Science Foundation of China (Project number: 81772700 and 81972400), the Beijing Capital Science and Technology Leading Talent Project (Project number: Z181100006318007), CAMS Innovation Found for Medical Sciences (Project number: 2019-I2M-1-003) and the China Postdoctoral Science Foundation (Project number: 2020M670221).

## Conflict of Interest

The authors declare that the research was conducted in the absence of any commercial or financial relationships that could be construed as a potential conflict of interest.

## Publisher’s Note

All claims expressed in this article are solely those of the authors and do not necessarily represent those of their affiliated organizations, or those of the publisher, the editors and the reviewers. Any product that may be evaluated in this article, or claim that may be made by its manufacturer, is not guaranteed or endorsed by the publisher.

## Supplementary Material

Supplementary Table 1 Quantitative RT-PCR primers.

Supplementary Figure 1
**(A)** The expression of circRAB3IP was detected in C4-2-EnzR and LNCaP-EnzR cell lines by qRT-PCR. **(B)** The expression of circUNC13B was detected in C4-2-EnzR and LNCaP-EnzR cell lines by qRT-PCR. **(C)** qRT-PCR analysis detected circRAB3IP expression in BPH (n = 10), l-PCa (n = 10), h-PCa (n = 10) and EnzR PCa (n = 6) tissues. **(D)** The expression of circRAB3IP was detected in h-PCa compared with l-PCa tissues by Yang dataset. **(E)** The expression of circRAB3IP was detected by MiOncoCirc database. Data are presented as mean ± SEM. *P < 0.05, ***P < 0.001 compared with the controls. N.S., not significant compared with the controls.

Supplementary Figure 2
**(A)** qRT-PCR assays demonstrating circRAB3IP levels after knocking down circRAB3IP in C4-2-EnzR and LNCaP-EnzR cell lines. **(B)** qRT-PCR assays demonstrating RAB3IP mRNA levels after knocking down circRAB3IP in C4-2-EnzR and LNCaP-EnzR cell lines. **(C)** qRT-PCR assays demonstrating circRAB3IP levels after overexpressing circRAB3IP in C4-2 and LNCaP cell lines. **(D)** qRT-PCR assays demonstrating RAB3IP mRNA levels after overexpressing circRAB3IP in C4-2 and LNCaP cell lines. **(E)** C4-2-EnzR cells were treated with different concentrations of Enz as indicated after knocking down circRAB3IP #2, and cell viability was evaluated by CCK-8 assay. **(F)** LNCaP-EnzR cells were treated with different concentrations of Enz as indicated after knocking down circRAB3IP #2, and cell viability was evaluated by CCK-8 assay. Data are presented as mean ± SEM. *P < 0.05, **P < 0.01 compared with the controls. N.S., not significant compared with the controls.

Supplementary Figure 3
**(A)** starBase v3.0 predict the binding sites of circRAB3IP with miR-133a-3p/miR-133b. **(B)** The RNA expression of circRAB3IP was detected by qRT-PCR after treatment with miR-133a-3p mimics and miR-133b mimics. **(C)** The expression levels of miR-133a-3p and miR-133b were detected by PCR after knocking down circRAB3IP in EnzR PCa cells. **(D)** starBase v3.0 predict the expressions of miR-133a-3p and miR-133b in TCGA-PRAD database. **(E)** GEPIA database predict the expressions of miR-133a-1 and miR-133b in TCGA-PRAD database based on T stage. **(F)** GEPIA database predict the expressions of miR-133a-1 and miR-133b in TCGA-PRAD database based on pathology N stage. Data are presented as mean ± SEM. N.S., not significant compared with the controls.

Supplementary Figure 4
**(A)** Analysis of RNA-seq data (GSE104935) from EnzR PCa cells, miR-133a-3p potential target gene data from Targetscan database and cancer genes from OncoKB database. **(B)** The protein levels of SGK1, PREX1, HOXA9 and PTPRK were detected by WB in C4-2 and C4-2-EnzR cells. **(C)** The protein levels of SGK1 were detected by WB in LNCaP, C4-2, 22Rv1 and C4-2-EnzR cells. **(D)** The protein levels of SGK1 were detected by WB in EnzR PCa samples (N=3) and EnzS PCa samples (N=3). **(E)** starBase v3.0 predict the binding sites of SGK1 with miR-133a-3p. **(F)** The protein levels of SGK1 were detected by WB after knocking down SGK1 in C4-2-EnzR and LNCaP-EnzR cell lines. **(G)** The protein levels of SGK1 were detected by WB after overexpressing SGK1 in C4-2 and LNCaP cells. Data are presented as mean ± SEM. *P < 0.05, **P < 0.01 compared with the controls. N.S., not significant compared with the controls.

Supplementary Figure 5
**(A)** The protein levels of eIF4A3 were detected by WB in EnzR PCa samples (N=3) and EnzS PCa samples (N=3). **(B)** The expressions of eIF4A3 were detected by WB after knocking down eIF4A3 in C4-2-EnzR and LNCaP-EnzR cell lines. **(C)** The expressions of eIF4A3 were detected by WB after overexpressing eIF4A3 in C4-2 and LNCaP cells. **(D)** Analysis of RNA-seq data (GSE104935) from EnzR PCa cells and 55 potential TFs of RAB3IP from PROMO database. **(E)** The protein levels of LEF1 were detected by WB in EnzR PCa samples (N=3) and EnzS PCa samples (N=3). **(F)** The expressions of LEF1 were detected by WB after knocking down LEF1 in C4-2-EnzR and LNCaP-EnzR cell lines. **(G)** The expressions of LEF1 were detected by WB after overexpressing LEF1 in C4-2 and LNCaP cells. **(H)** Site-directed mutagenesis of binding site #2 was done by mutating part of the sequence into Xba1 (–TCTAGA–) cutting site. **(I)** The expression levels of miR-133a-3p and miR-133b were detected by PCR after knocking down or overexpressing LEF1 in EnzR PCa cells. **(J)** The expression levels of miR-133a-3p and miR-133b were detected by PCR after knocking down or overexpressing eIF4A3 in EnzR PCa cells. **(K)** Ualcan database predict the expressions of eIF4A3, LEF1 and RAB3IP in TCGA-PRAD database. **(L)** Ualcan database predict the expressions of eIF4A3, LEF1 and RAB3IP in TCGA-PRAD database based on Gleason score. **(K)** Linkedomics database predict the expressions of eIF4A3 and LEF1 in TCGA-PRAD database based on T stage. **(M)** Linkedomics database predict the expressions of eIF4A3 and LEF1 in TCGA-PRAD database based on N stage. **(N)** GEPIA database predict disease free survival rate based on eIF4A3 or LEF1 expression in TCGA-PRAD database. N.S., not significant compared with the controls.
