# Supplementary material for: The circRAB3IP Mediated by eIF4A3 and LEF1 Contributes to Enzalutamide Resistance in Prostate Cancer by Targeting miR-133a-3p/miR-133b/SGK1 Pathway
Source: Front Oncol. 2021 Nov 15;11:752573. doi: 10.3389/fonc.2021.752573 (PMC8634431; doi:10.3389/fonc.2021.752573)
Supplement: Supplementary Table 1 — Quantitative RT-PCR primers. [file Table_1.docx]

**Table Quantitative RT-PCR primers**

| circRAB3IP | Forward | GTTCTGGAGGCTGTGGAAAA |
| --- | --- | --- |
|  | Reverse | GACACGTCCTGTCCATTGTG |
| circUNC13B | Forward | CAGCAGCGCAACATCTTCC |
|  | Reverse | CGGTCCTTAAACTCACGCCT |
| GAPDH | Forward | GGAGCGAGATCCCTCCAAAAT |
|  | Reverse | GGCTGTTGTCATACTTCTCATGG |
| RAB3IP | Forward | AAGCTGAAGTAGCTGCATTGAA |
|  | Reverse | GCCACTCATAGCACTGCTTGT |
| eIF4A3 | Forward | AAGGGAGAGATGTCATCGCAC |
|  | Reverse | GCTTGAGTTTCACGAACCTGA |
| LEF1 | Forward | AGAACACCCCGATGACGGA |
|  | Reverse | GGCATCATTATGTACCCGGAAT |
